# Development and Characterization of VEGF165-Chitosan Nanoparticles for the Treatment of Radiation-Induced Skin Injury in Rats

**DOI:** 10.3390/md14100182

**Published:** 2016-10-11

**Authors:** Daojiang Yu, Shan Li, Shuai Wang, Xiujie Li, Minsheng Zhu, Shai Huang, Li Sun, Yongsheng Zhang, Yanli Liu, Shouli Wang

**Affiliations:** 1 Department of Plastic Surgery, the Second Affiliated Hospital, Soochow University, Suzhou 215004, China; ydj51087@163.com (D.Y.); wsyy0514@163.com (S.W.); 18362720093@163.com (X.L.); 2 Department of Pathology, School of Biology & Basic Medical Sciences, Soochow University, Suzhou 215123, China; zhuminsheng714@126.com (M.Z.); hshine1992@126.com (S.H.); 20144221021@stu.suda.edu.cn (L.S.); 3 College of Pharmaceutical Science, Soochow University, Suzhou 215123, China; m18862163651@163.com; 4 Department of Pathology, the Second Affiliated Hospital of Soochow University, Suzhou 215004, China; shengyongzh@163.com; 5 Institute of Radiology & Oncology, Soochow University, Suzhou 215006, China; 6 Suzhou Key Laboratory of Tumor Microenvironment Pathology, Suzhou 215123, China

**Keywords:** radiation-induced skin injury, VEGF, chitosan, nanoparticles, apoptosis

## Abstract

Radiation-induced skin injury, which remains a serious concern in radiation therapy, is currently believed to be the result of vascular endothelial cell injury and apoptosis. Here, we established a model of acute radiation-induced skin injury and compared the effect of different vascular growth factors on skin healing by observing the changes of microcirculation and cell apoptosis. Vascular endothelial growth factor (VEGF) was more effective at inhibiting apoptosis and preventing injury progression than other factors. A new strategy for improving the bioavailability of vascular growth factors was developed by loading VEGF with chitosan nanoparticles. The VEGF-chitosan nanoparticles showed a protective effect on vascular endothelial cells, improved the local microcirculation, and delayed the development of radioactive skin damage.

## 1. Introduction

Radiotherapy is one of the standard treatment options for patients with cancer, as ionizing radiation can kill tumor cells by generating free radicals (FR) or reactive oxygen species (ROS). However, the beneficial effects of radiation are modest in the vast majority of patients. The failure of targeted radiotherapy lies in the non-discriminative killing of both cancer and normal cells. Radiation-induced skin injury is the most common complication, and late effects, which are the most severe, are characterized by subcutaneous fibrosis and morbidity [1,2]. Recent advances in cancer radiation biology resulted in the identification of new and promising targets for tumor radiosensitization in addition to normal tissue radioprotection in radiotherapy [3]. Growth factors have been used for a long time to rescue progenitor cells and hematopoietic cells following irradiation. Current research has focused on microcirculation, an important breakthrough in the treatment of radioactive skin damage with typical features of radiation-induced vasculopathies, including necrosis and inflammation within the arterial wall [4]. Therefore, reducing the damage to microcirculation and promoting the reconstruction of impaired microvessels are key issues in the treatment of skin radiation injury.

Vascular endothelial growth factor (VEGF), a master regulator of angiogenesis, has the ability to start a complex cascade of events leading to endothelial cell activation, assembly of new vascular structures, mural cell recruitment, and vessel stabilization [5,6,7]. However, incorporation of growth factors is difficult due to their short half-life of only several minutes in circulation [8]. Many studies have reported the development of VEGF-loaded nanoparticles for wound-healing angiogenesis, bone regeneration, or inhibition of the graft shrinkage [9,10,11,12]. Chitosan (CS) nanoparticles—as non-viral vectors that can deliver cytokines with low toxicity, favorable biodegradability, and lack of immunologic effects—have been studied extensively by our and other research groups [13,14,15,16]. Here, we successfully developed CS nanoparticles loaded with VEGF165, a powerful vascular growth factor [17], in a model of radiation-induced skin injury in rats. The results showed that the VEGF-CS nanoparticles protected vascular endothelial cells, improved the local microcirculation, and alleviated radiation-induced skin injury in rats.

## 2. Results and Discussion

### 2.1. The Characteristics of CS Nanoparticles Formulated with VEGF

VEGF has been formulated into various sustained release delivery systems because of its short half-life. The long-term VEGF delivery is usually based on its encapsulation in biodegradable polymers, which are designed to release the loaded VEGF in a sustained manner following the degradation of the polymer. To improve the sustained release, VEGF has been conjugated with CS, which secures the loaded growth factor and releases it in a biologically relevant manner. Here, we used CS nanoparticles prepared by the ionic interaction between a positively charged amino group of CS and a negatively charged counter-ion of tripolyphosphate sodium (TPP). CS has been used to improve or control drug release based on its ability to form a hydrogel spontaneously upon contact with multivalent polyanions. 

**Figure 1:**
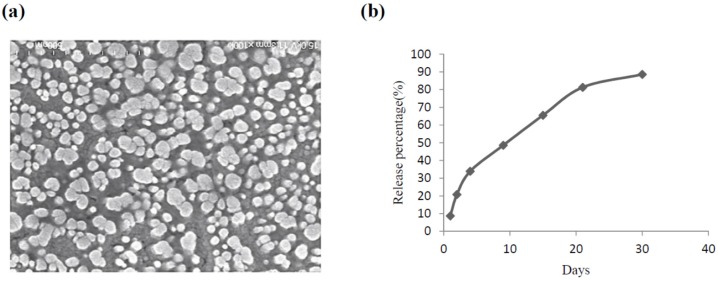
Characteristics of vascular endothelial growth factor (VEGF)-chitosan (CS) nanoparticles. (**a**) Representative shapes of VEGF-CS nanoparticles via scanning electron microscope (SEM) technology; (**b**) Release percentage of VEGF-chitosan nanoparticles in vitro.

VEGF-CS nanoparticles were prepared by adding a sodium tripolyphosphate solution to a chitosan solution under stirring as previous reported [15]. Figure 1a shows a representative scanning electron microscope (SEM) image of particles with spherical structures and almost uniform size distribution. The average particle size was 386.9 nm, the Zata potential was 25.3 V, and the encapsulation rate reached up to 85% (data not shown). VEGF was released from nanoparticles for 30 days. In the first 2 days, the amount of VEGF released was 10%–20%, with a gradual increase for more than 20 days (Figure 1b). This result demonstrated that CS nanoparticles achieve a controlled release of VEGF, suggesting that VEGF-CS nanoparticles are a promising delivery system for the treatment of radiation-induced skin injury.

### 2.2. Establishment of a Radiation-Induced Skin Injury Model

**Figure 2:**
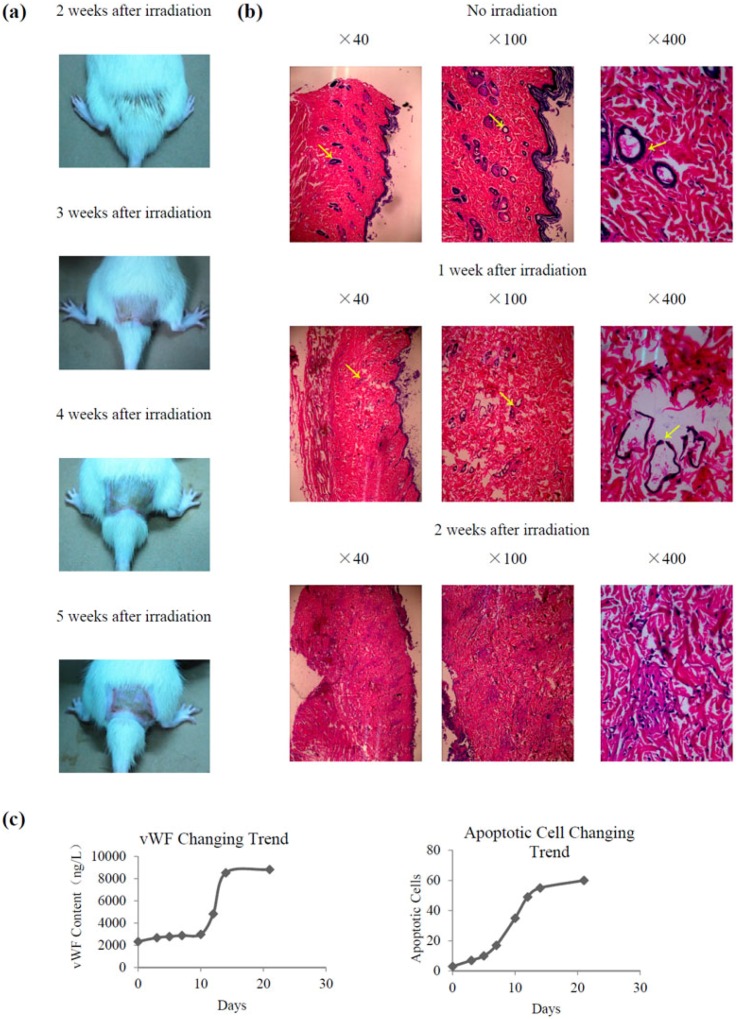
Establishment of a radiation-induced skin injury model. (**a**) Macroscopic images of radiation-induced skin injury in rats at 2–5 weeks; (**b**) Histological changes associated with radiation-induced skin injury in rats at 1 and 2 weeks after irradiation and in the control group; (**c**) Changes of von Willebrand factor (vWF) content and number of apoptotic vascular endothelial cells. Arrows pointing vessels.

All rats survived after receiving 45 Gy X-ray irradiation, with varying degrees of depression, reduced activity, and weight loss. Acute skin reactions were detected after 1 week of irradiation. Hair loss was detected at 2 weeks after irradiation, while local skin erythema, blisters, and eczema appeared at approximately 3 weeks after irradiation. Skin erosion, necrosis, and ulceration were observed at 4 weeks after irradiation, and chronic ulcers developed gradually starting at 5 weeks (Figure 2a). In the histological study, as shown in Figure 2b, injury can be found in the irradiated skin group. The number of vessels decreased after 1 week of irradiation, and inflammatory cells began to infiltrate. The vascular basement membrane was partly disintegrated and incomplete, and necrosis of endothelial cells and microthrombosis were observed. After 2 weeks of irradiation, the blood vessels disappeared, and a large number of inflammatory cells infiltrated the irradiation area.

Apoptosis of vascular endothelial cells and changes of von Willebrand factor (vWF), which closely resembled those of vascular disease, were a sensitive index of vascular endothelial cell injury [18,19,20]. Figure 2c shows that the contents of vWF and the number of apoptotic cells increased after 1 week of irradiation and nearly reached a peak after 2 weeks, then remained stable, with significantly higher values than those of the non-irradiated group (*p* < 0.05).

### 2.3. Screening of Vascular Growth Factor Treatment in Radiation-Induced Skin Injury

**Figure 3:**
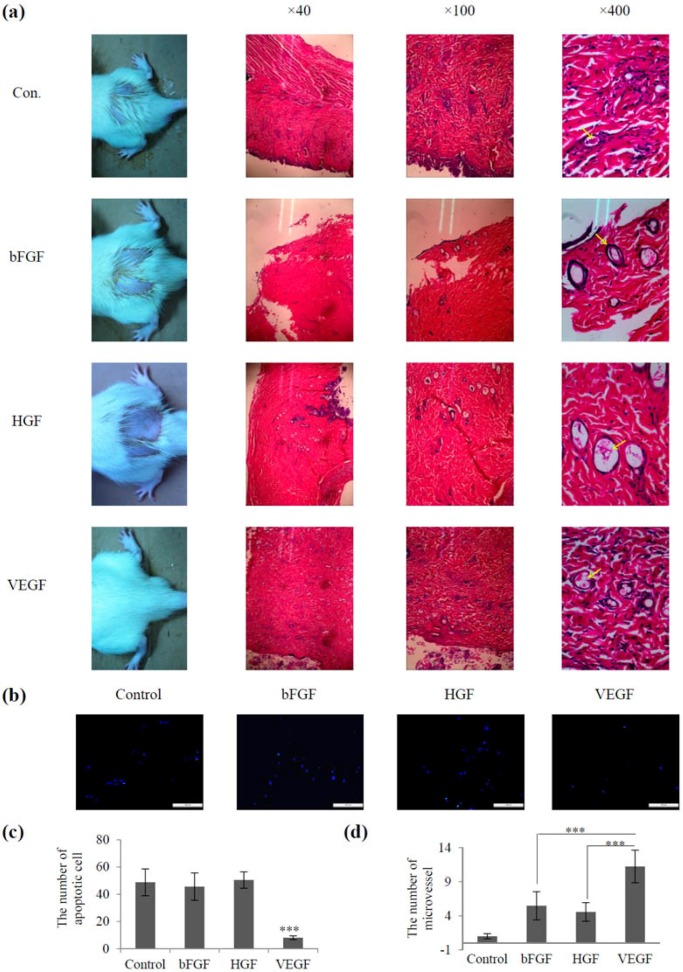
Screening of growth factor treatment for radiation-induced skin injury. (**a**) Macroscopic images and histological changes in irradiation-induced skin injury groups treated with different growth factors; (**b**) Representative images of apoptosis staining-positive cells upon basic fibroblast growth factor (bFGF), hepatocyte growth factor (HGF), and VEGF treatment (×200); (**c**) Quantification of apoptotic cells upon bFGF, HGF, and VEGF treatment (**d**) Number of microvessels in skin sections of all groups observed under the microscope. Data represent the mean ± SD from three independent experiments. *** *p* < 0.005 (**c**–**d**). Arrows pointing vessels.

The same amounts of growth factors such as basic fibroblast growth factor (bFGF), hepatocyte growth factor (HGF), and VEGF were used for radiation-induced skin injury treatment. Figure 3a shows that after 2 weeks of treatment, all rats displayed hair loss in the control group, which was treated with equal volumes of normal saline. The bFGF- and HGF-treated groups showed slight hair loss, while the VEGF-treated group had no significant hair loss. Furthermore, the development of hair loss and ulcers was delayed in the VEGF group compared with that in the other groups. Morphological studies showed that the VEGF-treated group showed a nearly normal strata structure, complete re-epithelialization, and few hair follicles, whereas the other groups showed thickening of the epidermal layer and poorly vascularized granulation tissue. In addition, the number of apoptotic cells was lower in the VEGF-treated group than in the other groups (Figure 3b,c). The number of microvessels was higher in the VEGF group than in the other groups, and the difference was statistically significant (*p* < 0.05) (Figure 3d). Taken together, these data suggested that the rats injected with VEGF had better results than the other groups. This observation coincides with the results of other research groups, who used EVGF165 for the treatment of wounds in diabetic mice [21].

### 2.4. Effect of VEGF-CS Nanoparticles on Healing of Irradiation-Induced Skin Injury

**Figure 4:**
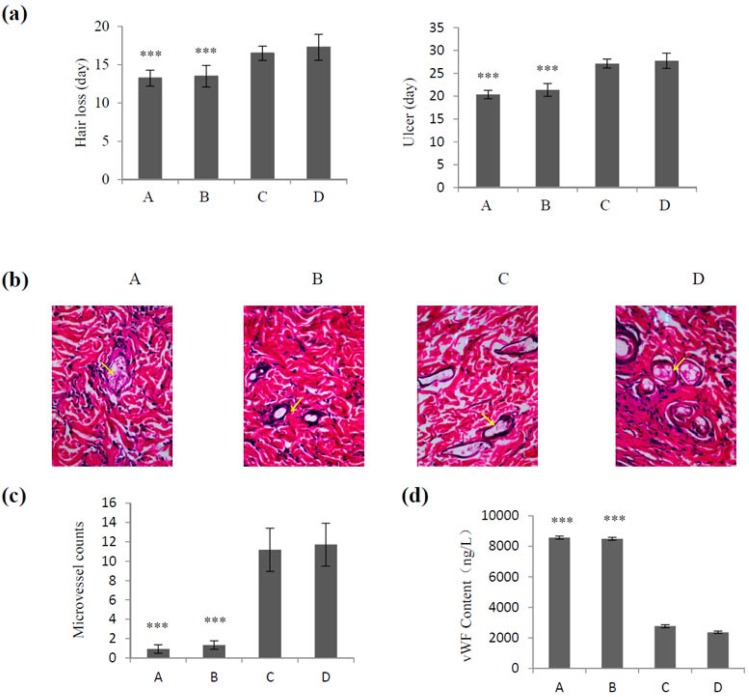
Effect of CS-VEGF nanoparticles on healing of irradiation-induced skin injury. The 64 rats bearing irradiation-induced skin injury were divided into four groups (A–D): A, single treatment with 1 mL normal saline as control; B, single treatment with 1 mL VEGF165 (700 ng/mL); C**,** treatment with 1 mL VEGF165 (100 ng/mL) daily for 1 week; and D, single treatment with 1 mL heavy suspension of VEGF165-CS nanoparticles. (**a**) Hair loss and ulcer development in each group after irradiation; (**b**) Hematoxylin and eosin staining (×100) of skin tissues; (**c**) Number of microvessels; (**d**) vWF content in each group. *** *p* < 0.005 (**c**–**d**). Arrows pointing to vessels.

VEGF165 promotes tissue repair in a rat model of radiation-induced injury [22] or relieves endothelial injury after deep vein thrombectomy [23]. In the present study, we explored the effects of different dose of VEGF165 loaded in CS nanoparticles and different delivery systems on irradiation-induced skin injury. As shown in Figure 4a, there were no differences in hair loss and ulcer development time between the group receiving a single treatment of VEGF165 (700 ng/mL) and the control group (injected with saline) (*p* > 0.05). However, when VEGF165 was injected daily (100 ng/mL) for 1 week, it delayed hair loss and ulcer development, and the difference was statistically significant compared with the control group (*p* < 0.01). A single injection of VEGF165 bound to nanoparticles (700 ng VEGF165) had a significantly better effect regarding the delay in hair loss and ulcer formation than that of the group receiving VEGF165 injection (*p* < 0.01). These results were confirmed by hematoxylin and eosin (HE) staining, which showed few microvessels in groups A and B, while rich microvessels were observed in groups C and D (Figure 4b). Figure 4c shows a significantly greater number of microvessels in the group treated with VEGF165 daily (100 ng/mL) for 1 week and that treated with a single dose of VEGF165 nanoparticles (loaded 700 ng VEGF165) than in the control group and the group receiving a single treatment of VEGF165 700 ng/mL (*p* < 0.01). These results were confirmed by measuring the content of vWF (Figure 4d) and suggested the potential value of VEGF-CS nanoparticles for the treatment of irradiation-induced skin injury.

### 2.5. Mechanism Underlying the Effect of VEGF165-CS on Alleviating Radiation-Induced Skin Injury

**Figure 5:**
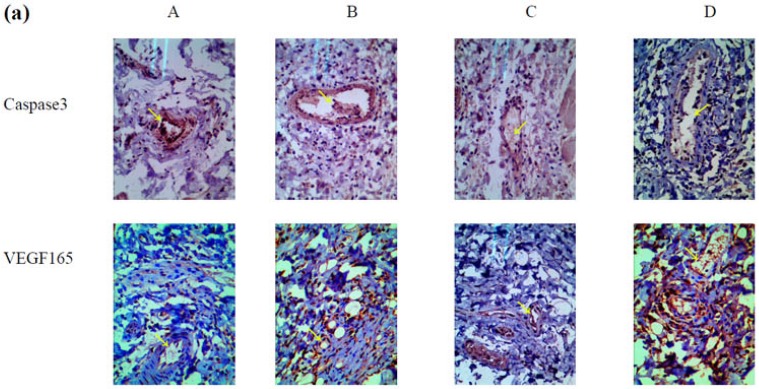

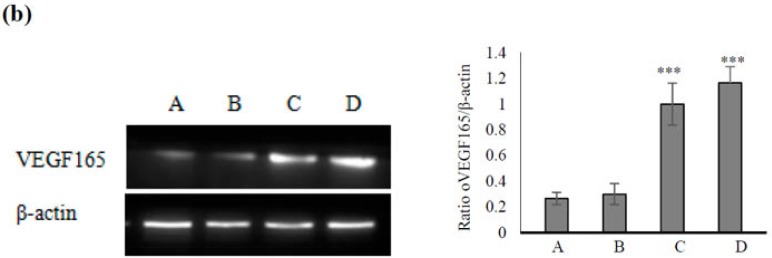
Mechanism of VEGF165-CS nanoparticle-mediated alleviation of radiation-induced skin injury. The 64 rats bearing irradiation-induced skin injury were divided into four groups (A–D): A, single treatment with 1 mL normal saline as control; B, single treatment with 1 mL VEGF165 (700 ng/mL); C**,** treatment with 1 mL VEGF165 (100 ng/mL) daily for 1 week; and D, single treatment with 1 mL heavy suspension of VEGF165-CS nanoparticles. (**a**) Immunohistochemical staining of caspase3 and VEGF165 in each group; (**b**) Western blot assessment of VEGF165 expression in each group (normalized to β-actin). Data represent the mean ± SD from three independent experiments. *** *p* < 0.005. Arrows pointing vessels or VEGF165 and caspase3 expression.

To explore the possible mechanism by which VEGF165-CS nanoparticles alleviated radiation-induced skin injury, we further examined the expression of VEGF165 and caspase3, which plays an important role in apoptosis in endothelial cells. As shown in Figure 5a, caspase3 expression was lower in groups C and D than in groups A and B. This result suggested that the effects of VEGF165 on inhibiting apoptosis and protecting endothelial cells in the form of nanoparticles were superior to those of a single injection and the control group. In addition, the expression of VEGF165 in groups C and D was significantly higher than that in groups A and B (Figure 5b). Furthermore, extensive vascular tissue and a complete lumen were observed in group D.

## 3. Experimental Procedures

### 3.1. Materials

Male Sprague Dawley (SD) rats (4 weeks old) were purchased from Shanghai SLAC Laboratory Animal Co., Ltd. (Shanghai, China). Chitosan with a degree of deacetylation of 95% and average molecular weight of 200 kDa, sodium tripolyphosphate, NaOH, and bFGF protein were obtained from Gibco (Shanghai, China). VEGF165 was purchased from Sigma-Aldrich (St. Louis, MO, USA). HGF was purchased from Invitrogen (Life Technologies, Carlsbad, CA, USA).

### 3.2. Methods

#### 3.2.1. The Establishment of a Model

Seventy-two healthy male rats weighting 220 ± 20 g SD were used. Rats were anesthetized by intraperitoneal injection of 10% chloral hydrate (0.35 mL/100 g). Rats were fixed with adhesive tape on a plastic plate. A 3 cm thick piece of lead was used to shield the rats and localize the radiation field (45 × 40 mm). A single dose of 45 Gy was administered to the buttock skin of each rat at a dose rate of 600 cGy/min using a 4 MeV electron beam accelerator (Phillips, Amsterdam, the Netherlands). To observe the hair and skin changes in the irradiated area, the animals were sacrificed and skin tissues and blood samples were collected. All animal procedures were approved by the Ethics Review Committee for Animal Experimentation of Soochow University.

#### 3.2.2. Enzyme-Linked Immunosorbent Assay (ELISA)

To detect the changes of vWF content, blood samples were analyzed by ELISA. Rats were sacrificed and blood samples were collected into citrated tubes, centrifuged at 3000 *g* (relative centrifugal force, RCF) for 10 min, and stored at 4 °C for subsequent analysis by ELISA according to the manufacturer’s data sheets (Shenggong, Shanghai, China).

#### 3.2.3. Cell Apoptosis Analysis

Cell apoptosis analysis was performed using the One Step TUNEL Apoptosis Detection Kit (Merck Millipore, Shanghai, China). Specimens were frozen and sections were fixed with 4% paraformaldehyde, and incubated in an ice bath. Then, the cells were stained with Hoechst 33258 (10 μg/mL) for 30 min. Nuclear condensation and fragmentation were observed under a fluorescence microscope (Eclipse TE2000-U, Nikon, Japan.)

#### 3.2.4. Screening of Vascular Growth Factors

The same approach was used to establish an irradiation-induced skin injury model as that described above. The injury animals were assigned to four groups (*n* = 16) and injected subcutaneously with 1 mL (100 ng/mL) HGF, VEGF, bFGF, or an equal volume of normal saline as control once a day for 1 week. The observation parameters were the same as those described above.

#### 3.2.5. The Preparation and Characterizations of CS Nanoparticles

CS nanoparticles were created by modified ionic gelation with negatively charged TPP ions [24]. Briefly, CS with a molecular weight of 200 kDa and a degree of deacetylation of 95% was dissolved to a concentration of 2 mg/mL in 1% acetic acid solution, and NaOH solution was added with slow stirring, followed by an equal volume of protein solution (20 ng/mL). TPP at a concentration of 1.5 mg/mL was prepared with deionized water and added dropwise under constant stirring to the mixture [25]. The physical size and zeta potential of the nanoparticles were measured using a 3000HSA Zetasizer (Malvern Instruments, Malvern, UK). The morphology of nanoparticles was observed by scanning electron microscopy (SEM).

#### 3.2.6. Release of VEGF from VEGF-CS Nanoparticles

The constant temperature oscillation method was used to measure VEGF association efficiency. CS nanoparticles (5 mg) and 3 mL PBS buffer were mixed in a 5 mL test tube. After constant shaking (100 r/min) at 37 °C, 3 mL of the supernatant were collected at different time periods and added with fresh medium at the same time. Then the release of protein content and the cumulative release rate of each time point were determined.

#### 3.2.7. Application of VEGF-CS Nanoparticles in Vivo

Sixty-four SD rats bearing irradiation-induced skin injury were randomly assigned to four groups according to the different VEGF165 delivery systems as follows: Group A, single injection of 1 mL normal saline only; Group B, single injection of 1 mL VEGF165 (700 ng/mL); Group C, injection of 1 mL VEGF165 (100 ng/mL) daily for 1 week; Group D, single injection of 1 mL suspension of VEGF165-CS nanoparticles (loaded 700 ng VEGF165). The observation parameters were as mentioned above.

#### 3.2.8. Hematoxylin and Eosin Staining and Immunohistochemistry

The skin specimens were fixed in 10% formalin for 2 days, embedded in paraffin, sectioned at 5 μm, and mounted. After the sections were stained with HE, the morphology and vascular density of skin tissues were observed under a light microscope (Olympus CX31, ×40 magnification). Immunohistochemistry staining was performed by the Streptavidin–Peroxidase kit method according to the manufacturer’s guide. In brief, paraffin sections were deparaffinized and incubated at 4 °C overnight with anti-VEGF165 and Caspase3 (Abcam, Inc., Cambridge, MA, USA) in PBS containing 1% BSA. Then, the slides were incubated with peroxidase-conjugated IgG (Shanghai Genomics, Shanghai, China) and counterstained with hematoxylin.

#### 3.2.9. Western Blot Determination of VEGF165 Expression

Skin tissues were harvested and lysed in TNES buffer. Equivalent 25 μg aliquots of proteins were electrophoresed and electrotransferred onto polyvinylidine difluoride membranes. After the blots were blocked, the membranes were incubated with 1:1000 anti-VEGF165 (Santa Cruz Biotechnology, Santa Cruz, CA, USA) for 3 h at room temperature, and visualized with peroxidase-conjugated secondary antibodies using an enhanced chemiluminescence detection system (Santa Cruz, CA, USA).

## 4. Conclusions

In the present study, we established a model of radioactive skin damage, and demonstrated that VEGF can inhibit apoptosis in vascular endothelial cells and accelerate wound healing in irradiated areas. However, the biological function of VEGF depends on its method of delivery. We developed a CS nanoparticle delivery system modified by TPP and successfully loaded VEGF165. Assessment of microvessels, vWF content, and apoptosis demonstrated that VEGF165-CS nanoparticles delayed the development of radiation-induced skin injury and promoted healing. The design of VEGF165-CS nanoparticles with controlled local release of VEGF165 for long periods provides an excellent delivery system for the treatment of radiation-induced skin injury.
